# The Effects of the War on the Mental Condition of the Citizens of Bristol

**Published:** 1920-06

**Authors:** R. H. Norgate

**Affiliations:** Medical Superintendent Poor Law Infirmaries, Bristol


### THE EFFECTS OF THE WAR ON THE MENTAL CONDITION OF THE CITIZENS OF BRISTOL.

BY

R. H. NORGATE, M.R.C.S., L.R.C.P.,

*Medical Superintendent Poor Law Infirmaries, Bristol.*

---

ALTHOUGH the Powers that be prevented me from, in Tisdall's words—

> "Wading through mud, and being spattered with blood,
> And living a week on three mice and a spud,"

yet they gave me the opportunity of being a spectator of one of the side-shows in that terrible drama of war—its effects on the minds of many of the citizens of Bristol. I have been a spectator with a dual personality: (1) as a medical man; (2) as a man trying to fathom the causes of their mental breakdown, and to record the effects of this struggle upon them.

The causes have been many: a victory, a reverse, trench warfare, stalemate, sudden social upheavals, rising of prices, air shocks, atrocities, periods of long waiting for news, lack of nourishment. In conclusion, the very slow return to what we cannot yet call the normal conditions, has left a sad jetsam of permanently disordered minds which will take years to collect, and a flotsam of shattered fragments tossed on a world which has no further use for them.

This paper deals with the mental cases under my care during the war at Stapleton and Eastville Institutions, those just before the outbreak of war, and those for some months after peace was signed. Yet this is no final conclusion, for nearly every day I am admitting cases whose mental breakdown is due directly or indirectly to the war.

In order to simplify my figures, I take from Lady Day, 1914, to Lady Day, 1920. Previous to the war, for some years past, there have been about 500 chronic lunatics in our mental wards. On March 25th, 1915, the Asylum was closed, and became Beaufort Hospital, and all the new cases were sent to me for observation at Stapleton, where special wards were set apart for their reception and treatment. Cases urgently requiring asylum treatment had to be sent to a series of asylums, eight in number, in the south-west, from Wells, the nearest, as far as Dorchester and Bodmin. It required careful consideration to send a very violent case as far as Bodmin, or even a very weak suicidal case by road to Wells. In addition, the police sent us all cases "found wandering." Patients were admitted at any time, night or day. But with the willing help of good and bright attendants, nearly all of whom had had asylum experience, we were able to keep the large majority of the cases, and especially the border-line cases. We eventually sent home "greatly improved in mind and body" a very large number of persons who were saved the anxieties and stigma of an asylum, and were able to be visited frequently by their friends or relations in Bristol. These wards were treated as mental wards of the hospital, and the inmates were treated as "sane persons" with reservations, ill in their minds. They responded rapidly to treatment, and, what was better, tried to influence others to improve also. This has made me confident that the asylum is not the place for border-line cases, and that every hospital should have wards set apart for this special purpose.

*Pre-war Excitement.*—It is of interest to notice that there was a great wave of warlike excitement in the world in the few months before the War. There was war in Mexico, almost civil war in Ireland, and the great suffragette rebellion, which led some few of them to step over the border-line of common sense and risk the loss of the respect which we owe to woman.

*Outbreak of War.*—Soon after the outbreak three events occurred which will always be present to my mind. (1) I saw the great fleet at anchor off Spithead. (2) I travelled up from Swansea to Bristol with the foreign reservists going home to join up. (3) I witnessed the departure of the Artillery from Whiteladies Road, in which in the old days I was a gunner in No. 6 Battery.

The effects of these three sights on my mind were strange. (1) Gave a feeling of absolute safety, a placid contentment, a "stuporous dementia" typically English. (2) A scene of maniacal excitement—cheering, shouting, flag-waving, hysterical outburst of Southern Europe—scenes repeated at every station on the line—followed later by intense melancholy when thoughts of home and family passed as visions across the mind—the brave behaviour of the mothers, the wives and the sweethearts—women at their best. (3) The deep silence, broken by the rumbling of the gun-limbers, the first view of modern engines of war. The solemn procession from the Drill Ground going forward "to some corner of a foreign field."

Whilst such scenes were in progress it was only natural that men, women, and children were affected somewhat differently. A wave of enthusiasm spread over the children of Bristol. A perpetual pantomime was being played for their special benefit all day long. School was out of the question, lessons were "nah poo," and battles were fought at every street corner. Sorrow and mental depression had begun to take the place of pride and excitement amongst the women, which was promptly checked by the suggestion of a food famine. Men were differently affected, and could be divided into three classes. (1) Those who foresaw what the struggle meant and what it might lead to, set their teeth and stood firm. (2) Those who looked upon it much in the light of a cup tie. (3) Those who at once began to take on a deep sense of injury against the Germans, and all foreigners—they hunted for spies, saw visions at night, and heard sounds of gun-fire, etc. These were people whose mental balance was already beginning to be shaky.

*Statistics.*—In my mental wards on March 25th, 1914, there were 546 certified lunatics, and during the six years 2,305 new patients were sent to me for mental observation.

| | *Male.* | *Female.* |
|---|---|---|
| Acute mania with delusions | 460 | 519 |
| Acute melancholia with delusions | 77 | 226 |
| Alcoholic mania | 55 | 37 |
| Epileptic mania | 56 | 44 |
| Puerperal mania | — | 16 |
| Erotomania | — | 23 |
| General paralysis | 51 | 10 |
| Simple dementia | 64 | 46 |
| Senile dementia | 213 | 224 |
| Mental deficients | 72 | 96 |
| War cases | 306 | 335 |
| Sent to asylum | 226 | 262 |
| Kleptomania | — | 2 |
| Shell-shock | 24 | — |
| Pneumonia mania | 5 | — |
| Not insane | 2 | — |
| Drug-takers | 1 | 1 |
| Myxœdema | 1 | 4 |
| Torpedoed cases | 3 | — |
| Foreigners | 5 | 6 |
| Deserters | 3 | — |
| Suicides | 2 | — |
| War nurses | — | 1 |

I find, by the courtesy of Dr. Blachford, that 172 persons were sent to the Asylum in the first year, 79 men and 93 women, of whom 23 men and 29 women were sent from our mental wards. The rest went direct to the Asylum after examination at the Police Court. The Asylum was then closed.

A total of 2,969 mental cases came under observation. Of these I find that 306 men and 335 women have been affected more or less by war troubles, and 226 men and 265 women have been sent to various asylums. The rest have been nursed in our mental wards.

It is interesting to note that there were only two weeks during the whole six years when there was no admission to the mental wards, July 6th and August 9th, 1919 ; and the highest number admitted in one week was 16 in the first week in July, 1915, following the great German attacks at Hooge and the issue of the heavy casualty lists.

The more important battles, and the huge casualty lists issued afterwards, naturally had their depressing effect ; but the Parliamentary Orders affecting " the supply of men necessary for the carrying on of the war to a successful issue " encouraged the women, for everybody would be served the same. The thought of this gave them some heart, and melancholia went down.

The report of lack of munitions very properly aroused the country to a high pitch. Mania was predominant. Zeppelin and air-raids acted in a contrary manner.

The Food Orders issued in due rotation came in for a torrent of abuse, and were a potent factor in the number of cases dealt with ; but the gradual elimination of articles of food was so necessary, that it seems almost unbelievable that the Food Controller was obliged to fix the price of horseflesh for human consumption.

*Stapleton Institution.*—In the first place I should like to draw attention to the patients who were already in the mental wards.

There was a rush of feeble-minded youths to abscond from the buildings in order to enlist in the army under Lord Derby's scheme. Of these one was told " they could not find a pair of boots big enough for his feet," and the majority were sent back on my representation. A few epileptics passed the doctor, and were afterwards returned.

Some old soldiers brightened up a little at first, recounted their battles, and then relapsed into their demented state.

General paralytics boasted as usual, and decorated themselves with home-made ribbons and medals.

Many of the women took a keen interest in the war, and were particularly elated if they had a relative at the front.

The war diets had a most disastrous effect upon them. Accustomed to eat practically as much as they liked, they were badly hit, lost flesh very rapidly, and suffered severely. We had repeated outbreaks of typhoid and a severe bout of dysentery Y bacillus.

*Early War Strain.*—There are a few points essential to remember in the early part of the War which affected the minds of the people. (1) The early lists showed casualties which were disconcerting by their numbers as compared with other wars. (2) Arrival of wounded men in Bristol. (3) The wounded men brought home bad accounts of the conditions in the trenches. (4) Effect on men who might be called up. Several who had been in the lunacy ward for alcoholism and epilepsy came at once for medical certificates. (5) Mental stability of the men examined was hardly tested. (6) Strain of training on men with a lower ratio of mental stability. (7) Men and women who had been previously insane broke down again, the delusions now assuming a warlike character.

*Alcoholism.*—I have listed 92 cases of alcoholic mania,

55 men and 37 women. The large majority of cases occurred in the first year. There was a remarkable diminution in 1917–1918, when Bristol went practically dry, and "the Government ale was really more a moral than a physical enjoyment."

The early days of war were responsible for some hilarity, and also for the downfall of some women. From January 1st, 1917, to April 29th, 1919, only one woman was admitted in this state needing to be certified.

*Sexual Mania.*—The sexual manias numbered 23 young women of varied classes, and nearly all of them had some hereditary mental history. These cases were often complicated with venereal disease, and several are likely to lapse into moral imbecility.

The greater freedom, the lax license allowed, the lack of supervision at home, and possibly the bad example set by the superior classes are responsible for this, and more particularly the eroticism in the modern films.

*General Paralysis of the Insane.*—61 cases, 51 men and 10 women. Early in the War German Emperors began to arrive, with large armies, fleets of aeroplanes and motor-cars, millions of money to spend—all on paper—cheques for thousands, but no small change in their pockets. His day is past and over for G.P.I.'s.

Later a new type arrived, much younger men, who had gone through some severe shock on land or sea. Some had not been out of England, but in "the air-raid" area; and others were sailors torpedoed at sea, with positive Wassermann's reaction and a history of syphilis acquired at about ten years' interval or less. The shock was too much for a brain already affected by syphilis. Their paralysis was expedited by some five to ten years.

Two young G.P.I.'s I have seen — a half-brother (a soldier) and his sister (a war worker) by the same mother,

21 and 24 years old—both gave positive Wassermann reactions and rapidly advancing symptoms.

*Senile Dementia.*—210 men and 224 women. Not so many as one would have expected. With better wages for the working classes, they have been enabled to keep their aged relatives at home. They have been very cantankerous, not appreciating the idea of rationing of food and coal. Margarine has been a sore trial to them. And when the man has been at the Front grandmother has been useful in the house to look after the children and tidy up a bit, especially if mother has been doing a little to add to her slender war allowance.

*Mental deficiency.*—In this class I have placed the idiots, the imbeciles and feeble-minded—72 males, 96 females.

With father away it was a difficulty to know what to do with the feeble-minded, who could do little or nothing to keep the home going, and very properly they were sent in for observation. It was essential to keep the girls off the streets, and the boys got into mischief with virtually no parental control.

Some of the histories are quaint. One youth on his mother's death dressed himself up in her clothes and tried to do the housework.

Many of the girls went out to service at minimum wages, had many places, wandered about at night and fell into the hands of the women patrols, and were handed over to us.

*Simple Dementia.*—64 men, 46 women. 20 men and 12 women dementia precox, family history in nearly every case.

When the urgent calling up of men was in force one youth was quite content to remain in bed with every comfort bestowed upon him and with the care of a hospital nurse for two years. He let his hair grow long like a woman, nursed an imaginary spinal complaint, and played his part remarkably well. With shorn locks and a sharp application of the battery he improved, but has since relapsed in his own home.

The drill instructors weeded out these cases in the early days of training. The women's morals required careful attention.

*The Delusional Manias and Melancholias.* — Mania: males 460, females 519. Melancholia: males 77, females 226.

We see here a great preponderance of women melancholias.

We sometimes ask who has had to suffer most, men or women. There has been a greater strain thrown on women. I often wonder if we men really appreciate what many of these poor women went through during the War in their own homes—the strain, the anxiety, and the suffering, the racket of children, the crying of babies, the perpetual worries about food, and the quiet hours of suffering and thinking, the sleepless nights and the haunting uncertainty.

> " 'Tis they who are in their own chambers haunted,
> By thoughts that like unbidden guests intrude,
> And sit down uninvited and unwanted,
> And make a nightmare of the solitude."

They are best described in this one short sentence: "They said little, but their eyes asked questions."

At least one-third of these melancholias were suicidal.

A peculiar feature to be noticed was that these war delusional cases were never general at the same time over the whole city. The city is divided up into certain areas or districts, for each of which there is a relieving officer. For ten days or a fortnight the officers in turn would bring groups of these women to be examined. On only one occasion do I remember as many as five relieving officers bringing in cases during the same week.

The spread of the melancholias amongst women at certain periods of life is interesting. The catamenial period was easily first, and in each decennial period beneath this diminished in frequency.

June to September, 1915, when lack of munitions, Zeppelin raids, and huge casualties were reported, and the whole of 1916 and 1917, were critical times for women.

There was a strange rest from October to December, 1915, due, I believe, to Lord Derby's scheme and calling up.

The most shocking cases of all were some of the Belgian refugees.

The delusions both in mania and melancholia have been warlike, and I find that they have closely followed the events in the War. The commonest type was persecution by Germans or foreigners, and " by the lady next door " hunting for spies. Then came gas shells and liquid gas. They imagined that gas had been introduced by their neighbours or enemies through the roof, through holes in the walls, or under the floor. There were several cases of attempted self-destruction by gas. In only one case was sulphur detected. People ran about at night nude or nearly so, and sometimes in the day, being haunted by Germans. Jumping from high windows appealed to some, not always with a view to self-destruction — aeroplanes suggested this.

There has been a great fascination for drowning by women ; and they have told me again and again (women mostly from Redcliff and Bedminster, wives of sailors and dockers) that for days they have visited the river to drown themselves, but could not summon up courage to do so, and came over to Stapleton for self-protection.

Many men and women have shut themselves up in their homes and not ventured out of doors, contenting themselves with reading the evening papers, have lost their appetites and taken next to no food, their general health has deteriorated until their brains were an easy prey to delusions of suspicion, voices and visions of relatives killed at the Front.

The influence of the food queues acted for both good and evil. Whilst taking them out into the open air, they met others who had had recent bereavements, and their own worries were redoubled ; and the long standing, poorly clad, in bad weather led to missing of meals and affected their brains prejudicially.

The religious maniacs looked upon their bereavements as a direct punishment from God for some past sin.

Many women neglected their homes and families very much, not always through intemperance, but as the result of stuporous dementia engendered by the War, and some soldiers had very sad home-comings.

The Coroner has kindly furnished me with the following particulars :—

*Cases of Suicide in the City and County of Bristol.*

| Year. | Male. | Female. | Total. |
|---|---|---|---|
| 1911 | 15 | 5 | 20 |
| 1912 | 15 | 4 | 19 |
| 1913 | 21 | 10 | 31 |
| 1914 | 14 | 6 | 20 |
| 1915 | 10 | 6 | 16 |
| 1916 | 14 | 7 | 21 |
| 1917 | 11 | 9 | 20 |
| 1918 | 11 | 5 | 16 |
| 1919 | 13 | 8 | 21 |

Puerperal melancholia, three wives of soldiers at the Front.

Comparing the state of mind of the men and women in Germany and England, Gustav Roeder states : " ' Talk about your so-called atrocities which our men are said to have committed in Belgium,' they said to me, ' it would be nothing in comparison with what our men would do in England, and we women want to be there too.' "

*Epilepsy.*—56 males and 44 females. These cases have been very dangerous indeed, violent, and in some cases homicidal. The War and the food question have upset them severely. They are gross eaters frequently, have strong religious instincts, these fits have been very severe and frequent, but the cases of *petit mal* which were frequently met with had occasioned many acts of violence and ill-doing, which had brought them within the hands of the Law.

*Shell-shock.*—This leaves me with this most important group of cases, of which I have listed 24.

We have admitted, cast off from the war hospitals and asylums, cases which have been most difficult to judge in an impartial manner.

I had one most severe case, which beggars description—a most horrible wreck, an absolute skeleton, nerves all awry. Sent back to the Front after his nerves had given way. He died suddenly whilst eating his dinner. Four cases I have returned to asylums, ordinary manias and not shell-shocks. Several I treated successfully with electricity and moral persuasion.

One well-known Welsh footballer went off suddenly when a motor taxi started with rather an unusual noise. He had been celebrating his return, and was quite well again in two days. One case broke down after a stormy voyage with scanty provisions coming home from India, and is still very dull and dense.

But I am afraid there has not been the training for work that is necessary in these cases. They remain in hospital, attended to by sympathetic nurses, until all inclination for work has disappeared from their minds. They are discharged into a field where work has to be looked for and kept, when obtained, and good work is expected for good wages. All initiation is lost, and they gradually drift to a low ebb, are easily led, and become the associates of a low class of scoundrels, who use them as tools for a variety of evil deeds; and when the Law holds them they make the excuse of shell-shock, or plead mental instability from decadent parents.

The asylum is not the place for this class, nor the mental wards of an institution, but a modified Borstal system under medical supervision, which should train them gradually in graduated employment, the proceeds of which should pay for their keep; and there they should remain isolated from bad surroundings till they have seen the errors of their ways. Special hospitals built in country districts with land around should be provided for the stuporose, where with skilled medical observation they should be gradually roused from their lethargy and made useful members of the community. I would have prepared by impartial medical men a national registration card of health for every man, woman and child.

There are two points which have never been sufficiently commented upon in their relation to the War and sanity.

1. The great advantage gained to the country by allowing the former militant suffragettes to take charge of the arrangements for women workers. It allowed of a body of educated women (who might have been mischievous, and who were previously unoccupied) falling into line with "the mere man," thus calming their minds with good honest work; and though at first they took their slips and cuttings, the result was a successful exhibition of well-thought-out plans, which meant that in the place of the rough but ready care of the orderlies we got the skill and devotion of a band of women whose memory will always be fresh in the minds of the men in all parts of the world, and whose tact and kind assistance saved many a sweetheart, wife and mother from a mental breakdown.

2. The garden allotments. Middle-aged men with sons at the Front were taught that they could do their part in providing food for their families and the State. That open-air exercise changed their view of life—their melancholias and vain regrets were passing shadows only—and it has fostered in them a pastime which has added years to their lives and proved an inestimable benefit to the country.

In conclusion, I would say that we must get these cases of war mental disorder as early as possible under treatment, and not allow them to drift day after day with their misty vision into that " no man's land," where the mind stumbles into the pitfalls of delusions and the dangers of orientation, aimlessly wandering in a fog of hallucination, exposing themselves to the cross fires of enemy and friend; and instead of building more asylums, let us erect bright, cheerful homes in the country places, where a month or two's residence in pleasant surroundings, free from care and worry, will turn their minds to better thoughts, and not subject them to those scenes so familiar in an asylum, where we see "the faculties decaying, as the tree stands stark and helpless whilst its green leaves fall."

